# Examining Primary Care Physicians' Intention to Perform Cervical Cancer Screening Services Using a Theory of Planned Behavior: A Structural Equation Modeling Approach

**DOI:** 10.3389/fpubh.2022.893673

**Published:** 2022-05-24

**Authors:** Zhiqing Hu, Yanjun Sun, Yuhao Ma, Kejin Chen, Ling Lv, Lingling Wang, Yuan He

**Affiliations:** ^1^Institute of Medical Humanities, Nanjing Medical University, Nanjing, China; ^2^School of Marxism, Nanjing Medical University, Nanjing, China; ^3^Department of Women's Healthcare, Changzhou Maternal and Child Health Care Centre, Changzhou, China; ^4^Department of Women's Healthcare, Nanjing Maternity and Child Health Care Hospital, Nanjing, China; ^5^Department of Psychology, Kangning Hospital, Rushan, China; ^6^Research Center for Social Risk Management of Major Public Health Events (Key Research Base of Philosophy and Social Sciences of Universities in Jiangsu), Nanjing Medical University, Nanjing, China

**Keywords:** cervical cancer, primary care physicians, theory of planned behavior (TPB), intention, structural equation modeling

## Abstract

**Background:**

Promoting cervical cancer screening (CCS) is undoubtedly effective in combating severe public health problems in developing countries, but there are challenges to its implementation. Understanding the factors influencing primary care physicians' intentions to provide CCSs to rural women is crucial for the future implementation of screening programs. The aim of this study was to assess the intentions of primary care physicians to provide cervical cancer screening services (CCSSs) to rural women and their determinants.

**Methods:**

This cross-sectional study included 1,308 primary care physicians in rural primary health care, and the data collection tool was developed based on the theory of planned behavior (TPB), which included demographic characteristics, the basic constructs of TPB, and the degree of knowledge of CCSSs as an extended variable of the TPB model. Structural equation modeling was used to analyze the relationships between each factor.

**Results:**

Pathway analysis found that TPB is an appropriate theoretical basis for predicting primary care physicians' intent to provide CCSSs (χ2/df = 2.234 < 3, RMSEA = 0.035, and SRMR = 0.034). Meanwhile, the structural equation model showed that attitude (β = 0.251, *p* < 0.001), subjective norm (β = 0.311, *p* < 0.001), perceived behavioral control (β = 0.162, *p* < 0.001), and knowledge level (β = 0.152, *p* < 0.01) positively predicted primary care physicians' intention to provide CCSSs.

**Conclusions:**

TPB model, with the addition of knowledge, was useful in predicting primary care physicians' intention to provide CCSSs for rural Chinese women. The findings of this study provide a reference for the government and hospitals to develop strategies to improve the intent of primary care physicians to provide CCSSs.

## Introduction

Cervical cancer is one of the two most common cancers with high mortality rates, and it has the second-highest incidence globally among female malignant tumors, only behind breast cancer (1, 2). According to the World Health Organization, most new cases of cervical cancer occur in developing countries, with limited global medical resources (3, 4). As the largest developing country with a large population, China has an enormous cervical cancer burden and disparities between different regions. Research has shown that in 2012, there were about 61,691 new cases of cervical cancer in China, and this number will continue to reach 93,500 by 2030 if the situation does not improve (5). As stated by the International Agency for Research on Cancer, the cervical cancer screening (CCS) program is an effective strategy to address its incidence and mortality (6, 7), and the prevalence of cervical cancer in developed countries has significantly decreased with well-established screening programs (8, 9). Since 2009, the Government of China has launched NCCSPRA, a free National Cervical Cancer Screening Program in Rural Areas, which provides free cervical cancer screening services (CCSSs) to rural women aged 35 to 64. Despite the initiatives taken by the Chinese government, there were still many women in rural China who were either underscreened or never examined. Research conducted in 2011 found that the CCS rate in China was only 21.4% (10), which is significantly lower than in Finland (79.2%) (11) and Spain (65.6%) (12). Without appropriate action, cervical cancer continues to be a serious health concern that threatens the health and lives of rural Chinese women.

During the implementation of the screening program, rural women's general perceptions of CCS and their actual screening behavior can be changed by primary care physicians (13). Several studies (14, 15) have found that suggestions from primary care physicians can promote women's participation in cancer screening. One study (16) found that physician recommendations are an important predictor of patient mammography use; Grady (17) suggested that women would be more willing to participate in breast cancer screening programs with physicians' encouragement. The participation of Chinese women in a quantitative study (18) indicated that none of them had received any suggestions or information on the CCS from primary care physicians, hindering their participation in the CCS. All these studies have demonstrated the need to explore primary care physicians' intentions to provide CCSSs to rural women. However, most previous studies (19, 20) have focused on women's intention in CCS and few have addressed the factors that influence screening services provided by primary care physicians. Therefore, it is necessary to investigate the predictors of intention to provide CCSSs among primary care physicians in rural areas.

Ajzen's theory of planned behavior (TPB) (21) is a widely used social cognitive theory. TPB has been successfully used in different populations (22, 23), especially among primary care physicians, to understand the potential motivations for behavior. For example, Guibo (24) demonstrated the TPB's ability to understand the intention of Spanish nurses to use physical restraints, and Rich's research (25) revealed the efficacy of TPB in explaining medical physicians' behaviors. All of these studies have supported the utility of TPB in exploring the factors associated with behavioral intention among primary care physicians. Meanwhile, evidence-based research and meta-analyses (26, 27) have shown that TPB has more accurately defined constructs and greater explanatory power than other psychological theories or models such as HBM and TRA; therefore, in this study, it was hypothesized that the TPB could be a fundamental framework for identifying key determinants of providing CCS behavior among primary care physicians who have worked in rural areas in China. The results of the current study are expected to provide useful suggestions for improving CCS intent among primary care physicians, and policy recommendations for CCS program implementation can be developed based on the findings.

## Research Model and Hypothesis Development

**Figure 1:**
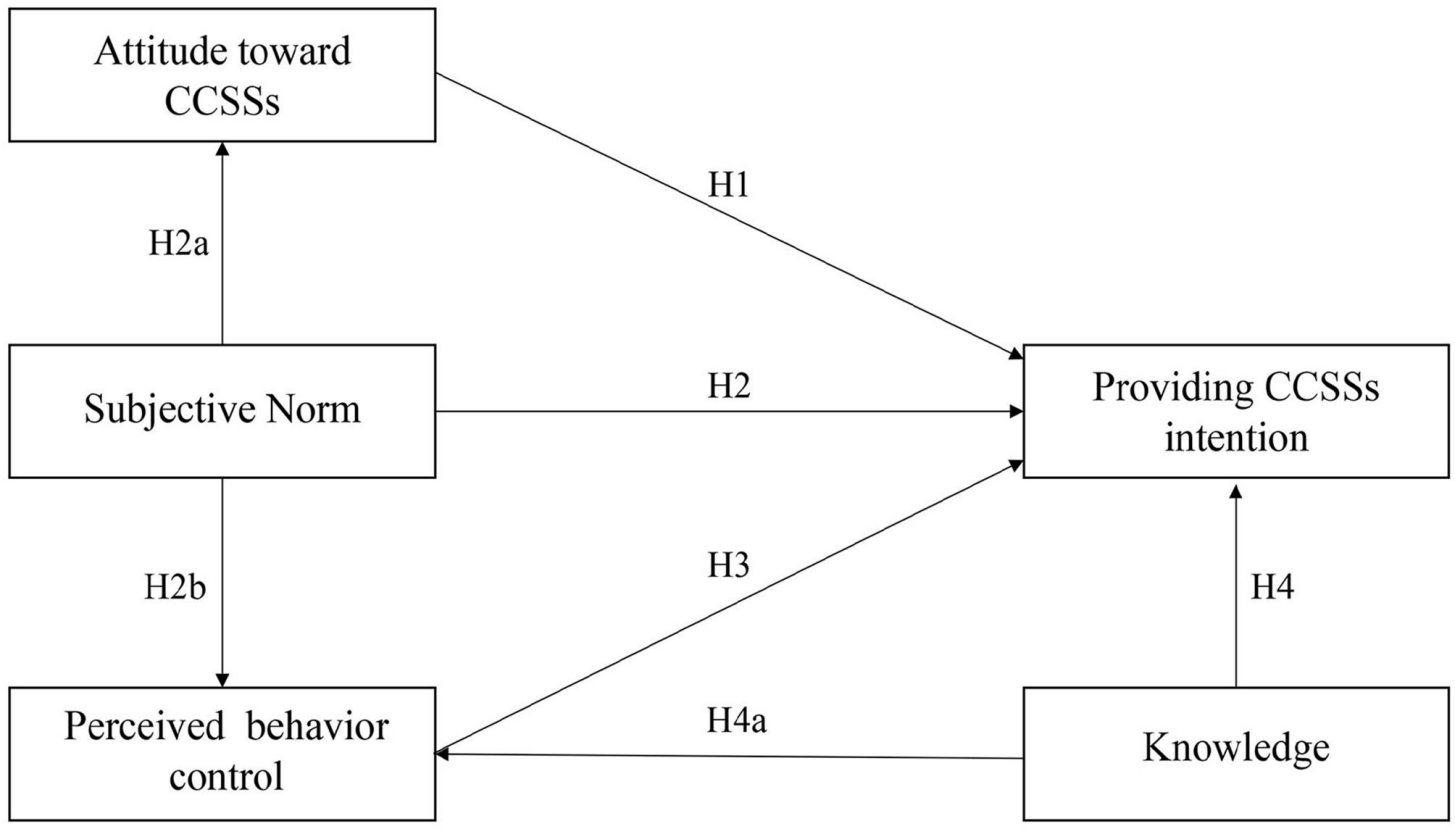
The research model of primary care physicians' providing CCSSs intention.

**Figure 2:**
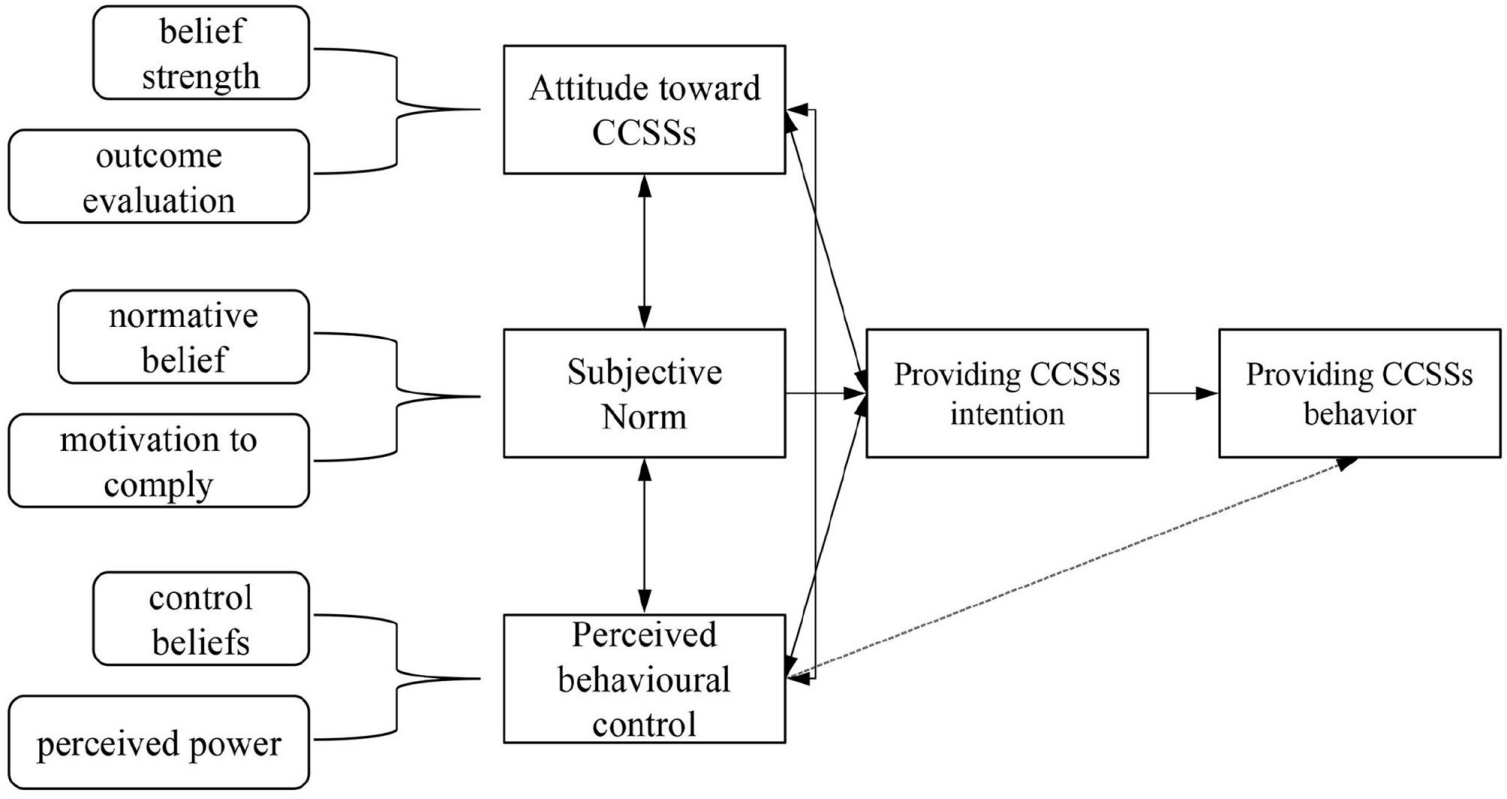
A Structural model of the theory of planned behavior.

A research model and related hypotheses (Figure 1) were developed based on the TPB and existing literature. According to the TPB (21), the intention is the proximal psychological predictor of individual behavior and is determined by attitude toward behavior (AB), subjective norms (SN), and perceived behavior control (PBC). Attitude toward behavior (in this case, primary care physicians' behavior of providing CCSSs) refers to the degree to which a person has a favorable or unfavorable evaluation of its performance (21), and is shaped by two components: behavioral beliefs (b) and outcome evaluation (e) of the behavior, which can be expressed by the following equation: AB = ∑*b*_*i*_*e*_*i*_ (i refers to the measurement project) (21, 28). SN refers to an individual's estimation of the attitude toward the behavior of their significant others. Similar to AB, SN is also determined by two distinct factors: normative beliefs (n) and motivation to comply (m) with normative beliefs, and the equation to evaluate SN is as follows: SN = ∑*n*_*i*_*m*_*i*_ (i means the measurement project) (21, 28). PBC refers to the degree of acceptance of an individual's perception of the performance of the behavior and includes control beliefs (c) and perceived power (p), as shown in the equation: PBC = ∑*c*_*i*_*p*_*i*_ (i means the measurement project) (21, 28). The research model is virtualized in Figure 2.

In this study, AB referred to primary care physicians' evaluation of the CCSSs outcomes. As recognized in TPB, AB is the most significant indicator of BI (21), and several previous studies have shown that attitude plays an important role in predicting primary care physicians' intention to provide medical services. A study conducted by Kim et al. (29) showed that attitude was a determinant of nurses' intention to provide medical care for SARS patients. At the same time, research conducted by Galaviz et al. (30) found that Mexican physicians' attitudes affect their intentions to prescribe physical activity (PA). Based on the TBP model and prior studies, the following hypothesis was proposed:

*Hypothesis 1: Attitude is positively associated with primary care physicians' intention to provide CCSSs*

In the present study, SN arises from the perception of primary care physicians as to whether their leaders, peers, and patients are applying the idea that they should provide CCSSs to rural women. In the TPB model (21), SN affects an individual's BI and is associated with AB and PBC. In a recent study, Nantha et al. (31) found that subjective norms can affect primary care physicians' intention to provide sick leave to patients. Herbert et al. (32) applied the TPB model in the context of clinical service behavior, indicating that physicians' intention to provide medication therapy management services (MTMS) was affected by their opinions on this service. Moreover, the opinion of colleagues on CCSSs can change the attitudes of primary care physicians. Administrators, policymakers, and the general public's acceptance of CCSSs can help them remove barriers to providing CCSSs for rural women. Therefore, the following hypothesis was proposed:

*Hypothesis 2: Subjective norm is positively associated with primary care physicians' intention to provide CCSSs*.
*Hypothesis 2a: Subjective norm affects primary care physicians' attitude toward CCSSs*.
*Hypothesis 2b: Subjective norm affects primary care physicians' PBC of providing CCSSs*.

The current study referred to the ease of providing CS services by a primary care physician. According to the TPB model, PBC affects BI (21). Previous research (33) has demonstrated that PBC is an indicator of physicians' intentions to provide clinical pharmacy services. A similar result was found in a study by Frankfurter et al. (34) and Liu et al. (35). Based on previous literature, we formulated the following hypothesis:

*Hypothesis 3: PBC is positively associated with primary care physicians' intention to provide CCSSs*

Studies (36, 37) have shown that the predictive utility of TPB can be increased by adding new variables such as knowledge level. General knowledge has been identified as a potential predictor of primary care physicians' intention to provide clinical services. On one hand, several studies (38, 39) have shown that knowledge about cancer screening was significantly and positively associated with primary care physicians' intention to provide CCSSs, and research on the application of TPB (40, 41) also revealed a link between knowledge and PBC. On the other hand, physicians who have sufficient CCS knowledge are more likely to understand the benefits of CCSSs and would have a more positive attitude toward CCSSs. Studies (42) have also found an association between knowledge and attitudes. Moreover, since the classification of medical and financial resources existed in different regions, lack of knowledge has become a common problem that makes it harder for primary care physicians to provide CCSSs (43). Subsequently, we added the knowledge level to the research model, which may have a potential effect on primary care physicians' BI and PBC of providing CCSSs, and the following hypotheses were posited:

*Hypothesis 4: knowledge of CCSSs has a positive impact on primary care physicians' intention to provide CCSSs*.
*Hypothesis 4a: knowledge of CCSSs is positively associated with primary care physicians' PBC*.
*Hypothesis 4b: knowledge of CCSSs is positively associated with the attitudes of primary care physicians*.

## Methods

### Sampling and Data Collection

In this cross-sectional study, multi-stage stratified sampling was carried out to select samples in rural areas of Jiangsu. In the first stage, six (Lianyungang, Yancheng, Yangzhou, Nanjing, Changzhou, and Wuxi) out of 13 cities in Jiangsu province were selected based on location and level of economic development. Two counties from each selected district and two towns from each selected county were then randomly chosen. Thus, 26 towns were selected for this study. A convenience sampling method was used to select participants in the 26 towns, who were primary care physicians working in public health care institutions. Those who were either sick or incapable of responding were excluded from this study. The minimum sample size was computed using Raosoft (www.raosoft.cpm/samplesize.html) (44) with a confidence level of 95%, margin of error of 5%, and a response distribution of 50%; the recommended sample size was 384. All data were collected between March 30^th^ and June 1st, 2020.

### Questionnaire

The questionnaire was adapted from the TPB model (21) and previous studies (28, 30, 45). To compile the questionnaire, first, several in-depth interviews based on the literature and the TPB model were conducted with rural primary care physicians who worked in different areas to explore the specific attitude, subjective norms, PBC, and intentions toward CCSSs. A pilot study was also conducted to evaluate the cultural sensitivity of the questionnaires, and a small group of people (including several experts and 30 primary care physicians) were asked to complete and assess the entire questionnaire. Each participant was also expected to revise the wording, phrasing, and overall construct. Based on the participants' feedback, a few modifications were made to improve the instruments' validity and reliability. Two measurements were included in the formal survey instrument: the sociodemographic characteristics of primary care physicians (e.g., gender, monthly income, level of education) and the CCSSs behavior intention questionnaire. The latter contained five subscales: attitude toward CCSSs (six items), SN (six items), PBC (eight items), behavioral intention to provide CCSSs (three items), and knowledge level of CCSSs (five items). The questionnaire was constructed as follows:

#### Attitude Subscale

The attitude was evaluated by multiplying two components: behavioral belief and outcome evaluation. There were three items that were used to measure behavioral beliefs (e.g., “I think providing CCSSs for rural women can save the cancer treatment costs”) with a five-point rating scale ranging from 1 (strongly disagree) to 5 (strongly agree). Other items were used to evaluate outcomes; for example, the item “I think saving the cancer treatment costs is” was followed by a rating scale ranging from 1 (not necessary at all) to 5 (very necessary). The overall attitude score was calculated by computing behavioral beliefs with the outcome evaluation, and a higher score indicated that primary care physicians had a more positive attitude toward performing CCSSs.

#### Subjective Norm Subscale

The SN was calculated by multiplying the products of “normative beliefs” and “motivation to comply”. Normative beliefs were measured by three items (e.g., “I think that most of my colleagues support me to provide CCSSs”), with a five-point rating scale ranging from 1 (strongly disagree) to 5 (strongly agree) as response options. Three items were used to evaluate the physician's motivation to comply (e.g., “Overall, I usually follow the suggestions given by my peers”) with a five-point rating scale ranging from 1 (strongly disagree) to 5 (strongly agree). After multiplying these two products, it was found that higher scores were closely associated with a higher SN.

#### Perceived Behavioral Control Subscale

According to the TPB model, the PBC subscale is measured by multiplying two components: controlled belief and perceived control. Three items were used to examine the control belief (e.g., “I think, the equipment of our hospital is”) and the response options ranged from 1 (don't have it at all) to 5 (very sufficient). Perceived control was also evaluated by three items (e.g., the item “I think, lacking equipment will make my CCS work”). A response scale ranging from 1 (very difficult) to 5 (not difficult at all) was the response option for the perceived control items. After multiplying these two components, it was concluded that the higher the scores, the higher the PBC of primary care physicians in performing CCSSs.

#### Behavior Intention Subscale

BI toward CCSSs was measured by three items, such as “I plan to provide CCSSs,” “I am willing to provide CCSSs” and “I try to provide the CCSSs,” the response scale ranging from 1 (strongly disagree) to 5 (strongly agree) with higher scores indicating that physicians have greater intention to engage in CCS program.

#### Knowledge Subscale

Five questions were designed to evaluate primary care physicians' knowledge level of CCSSs, including potential risk factors, screening methods and symptoms of cervical cancer. A two-dimensional scale was used in these questions (right = 1, wrong = 0), with possible overall scores ranging from 0–5. Higher scores indicated that primary care physicians had sufficient knowledge about CCS.

### Data and Statistical Analysis

The hypothesis model was analyzed by structural equation modeling (SEM) using Amos version 23.0 (IBM SPSS Amos, Armonk, NY, USA), which is a sophisticated statistical technique suitable for theoretical testing and has been widely applied in various scientific fields. First, exploratory factor analysis (EFA) was conducted using principal axis factor (PAF) analysis, and all data were screened by Kaiser–Meyer–Olkin (KMO) test of sampling adequacy and Bartlett's test of sphericity. Second, confirmatory factor analysis (CFA) was performed to conduct a satisfactory measurement model, and construct validity, convergent validity, and discriminant validity were evaluated to test whether the samples matched the theoretical model. Third, SEM was used to analyze the hypothesized research model and relationships among the variables. The fit indices for the model included the chi-square value of minimum sample/degree of freedom (CMID/DF), root mean square residual (RMR), standardized RMR (SRMR), root mean square error of approximation (RMSER), normed fit index (NFI), comparative fit index (CFI), and Tucker-Lewis index (TLI). The model was considered suitable for the samples as long as the following thresholds were met: χ2/df <3, CFI > 0.90, NFI > 0.90, TLI > 0.90, SRMR <0.08, and RMSEA <0.05 (46).

### Ethics Approval

This study was approved by the Ethics Committee of Sir Run Run Hospital, Nanjing Medical University. The grant number is 2019-SR-017. All participants provided verbal informed consent.

## Results

### Participants' Profile

In total, 1,308 primary care physicians were asked to complete a self-assessed questionnaire. After removing the invalid and incomplete responses, 1,120 valid questionnaires were finally obtained (valid response rate, 85.6%). As recommended by Bagozzi and Yi (47), the number that was used was considered adequate for further SEM analysis. The demographic profile of respondents is presented in Table 1. Most primary care physicians (91.3%) were women, whereas only 8.7% were men. Income differences among primary care physicians were evident: approximately 407 (36.2%) primary care physicians' salary was 3,000–5,000 RMB, 475 (42.4%) were paid 5,000–8,000 RMB, and only 11% received over 8,000 RMB per month. In terms of years of practice, primary care physicians' duration of work experience was long; approximately 45.8% of them had worked in hospitals for 20 years or more. A total of 475 (62.6%) primary care physicians had an undergraduate degree, indicating a high level of education. Almost half (43.7%) were employed in township health centers and 6.3% in rural maternal and child health centers.

**Table 1:** Demographics and relevant characteristics of participants (*n*=1120).

**Demographic variables**	**Frequency (*N*)**	**Percentage (%)**
Gender		
Male	97	8.7
Female	1023	91.3
Monthly income (RMB)		
≤ 3000	115	10.3
3000–5000	407	36.3
5000–8000	475	42.4
≥8000	123	11
Year of practice		
3 or less	62	5.5
4–10	218	19.5
11–20	316	28.2
>20	513	45.8
Level of Education		
Master	20	1.8
Bachelor	701	62.6
Associate degree	313	27.9
Others	86	7.7
Type of hospital		
Township Health Center	489	43.7
Village clinic	65	5.8
Rural Community Health Center	407	36.3
Rural Maternal and Child Health Center	70	6.3
Other	89	7.9

### Descriptive Analysis

The participants in this study showed a relatively positive attitude toward providing CCSSs to rural women; the average mean score of the three items used to evaluate the attitude of primary care physicians was 19.27 ± 4.45, with a positive response rate of 83%. For the variable SN, the average mean score was 17.76 ± 3.99, and the average positive response rate was 81.8%, both lower than the variable attitude, indicating that the participants in this study did not feel very well-supported when doing the CCS work. Among the TPB key variables, primary care physicians scored the lowest for the PBC variable (average mean = 12.11 ± 3.62, positive responses = 16.7%), meaning that the resources available to the participants were insufficient to enable them to provide CCSSs to rural women. The participants in the study showed a strong intention to provide CCSSs to rural women, with an average mean score of 4.14 ± 0.59 (with a possible score ranging from 0–5) and an average positive response rate of 79.2%. In terms of knowledge level, the primary care physicians in this study showed a satisfactory level of knowledge of CCSSs, with an average mean score of 3.85 ± 1.11 (with a possible score ranging from 0–5), and approximately 67.5% of the total knowledge score for primary care physicians reached a minimum of 4. The question regarding initial screening methods of CCS had the lowest correct ratio; only 47.8% of primary care physicians answered it correctly.

### Instrument Reliability and Validity

In this study, half (*N*=560) of the original data were used for exploratory factor analysis (EFA). The KMO test and the Bartlett sphericity test were performed to determine whether the questionnaire was suitable for factor analysis. Results suggested that KMO = 0.855 > 0.7, with a significant Bartlett test of sphericity (*p* <0.001) was suitable for the validity estimate (48). The maximum variance method was used to rotate all TPB factors (including attitude, SN, PBC, and BI), and the results showed that all factor eigenvalues were > 1 and the factor load for each item was > 0.5, indicating that the scale of the four-factor questionnaire can be well-explained by the measurement items. These four factors explained 19.849, 19.158, 18.298, and 17.517% of the variation, respectively, and the cumulative variance contribution rate was 74.823%.

Based on the sample of 560, the CFA was conducted to analyze the measurement model. The fit indices of the TPB model were as follows: χ2/df = 1.692 <3, RMSEA = 0.035 <0.05, SRMR = 0.030 <0.08, CFI = 0.990 > 0.9, TLI = 0.986 > 0.9 and NFI = 0.975 > 0.9, all of these indices were acceptable. The factor loadings of all items exceeded the recommended threshold of 0.5 for convergent validity (49), and the CR and AVE of each construct also exceeded the recommended threshold of 0.7 and 0.5 (50), respectively (Table 2). In addition, discriminant validity was found to be acceptable when the AVE of each construct exceeded the absolute correlation value for that construct (49) (Table 3).

**Table 2:** Convergent validity test (*n* = 560).

**Variables**		**Factor loading**	**CR**	**AVE**
AB	AB1	0.642	0.837	0.635
	AB2	0.873		
	AB3	0.854		
Subjective norm	SN1	0.833	0.744	0.897
	SN2	0.888		
	SN3	0.864		
Perceived behavioral control	PBC1	0.732	0.803	0.508
	PBC2	0.771		
	PBC3	0.755		
	PBC4	0.577		
Behavior intention	BI1	0.840	0.891	0.733
	BI2	0.940		
	BI3	0.781		

*AB, attitude toward behavior; SN, subjective norm; PBC, perceived behavior control; BI, behavior*.

**Table 3:** Discriminant validity test (*n* = 560).

**Variable**	**Attitude**	**SN**	**PBC**	**BI**
Attitude	**0.797**			
SN	0.651***	**0.866**		
PBC	0.248***	0.3357***	**0.713**	
BI	0.506***	0.512***	0.417***	**0.856**

*SN, subjective norm; PBC, perceived behavioral control; BI, behavior intention.*

*Diagonals (in bold) represent the square root of the AVE.*

*** *p <0.001*.

### Test of Structural Equation Model

**Figure 3:**
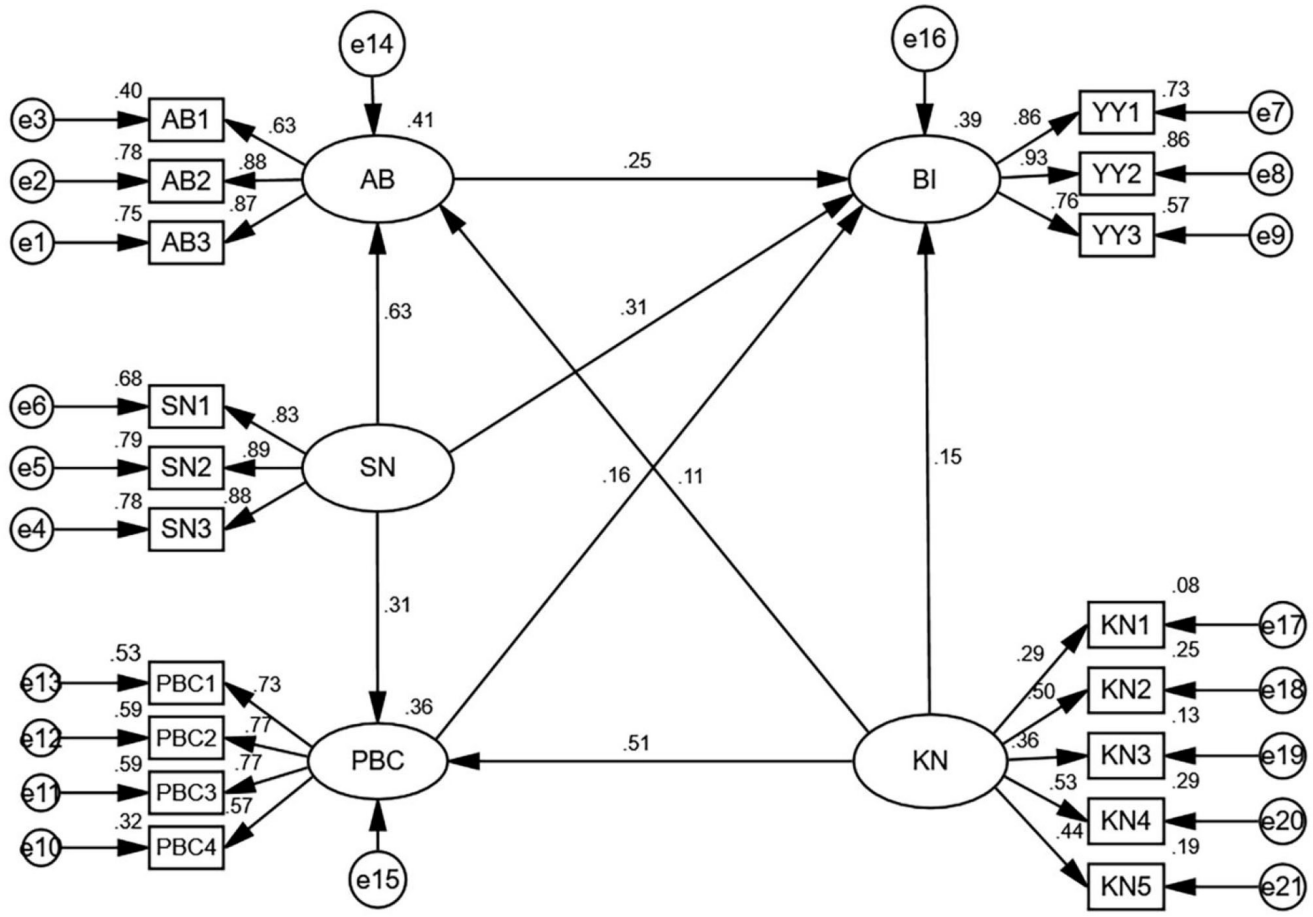
The Results of SEM analysis (*n* = 1,120). AB, attitude toward behavior; SN, subjective norm; PBC, perceived behavioral control; BI, behavior intention; KN, knowledge.

As the TPB based measurement model was accepted, the final model was built on the basis of the TPB variables and the knowledge factor for cervical cancer. The fit parameters for the extended model are as follows: χ2/df = 2.234 <3, RMSEA = 0.033 <0.05, SRMR = 0.034 <0.08, CFI = 0.981 > 0.9, TLI = 0.978 > 0.9 and NFI = 0.967 > 0.9. All indices fall within the appropriate range, indicating a good fit between the data and the theoretical model. A final structural model with the estimated standardized coefficients is shown in Figure 3, and the estimation results of the hypotheses presented in Table 4 show that they were all supported. As indicated by the results, an attitude in favor of CCS was associated with higher intentions to provide CCSSs (β = 0.251, *p* <0.001); SN of providing CCSSs was significantly associated with primary care physicians' intentions (β = 0.311, *p* <0.001), attitude (β = 0.630, *p* <0.001), and PBC (β = 0.309, *p* <0.001), and greater PBC was linked to a higher intention to provide CCSSs (β = 0.162, *p* <0.001). The knowledge level of CCS was significantly and positively associated with primary care physicians' BI to provide CCSSs (β = 0.152, *p* <0.01), as well as a predictor of attitude (β = 0.109, *p* <0.01) and PBC (β = 0.510, *p* <0.001).

**Table 4:** Results of structural equation modeling analysis.

**The hypothesis (H)**	**S.E**.	**C.R**.	**Estimate**	***p***	**Supported**
H1:Behavior intention ← Attitude	0.005	6.384	0.251	***	Yes
H2:Behavior intention ← Subjective norm	0.006	7.564	0.311	***	Yes
H2a:Attitude ← Subjective norm	0.035	19.876	0.630	***	Yes
H2b:Perceived behavior control ← Subjective norm	0.017	8.858	0.309	***	Yes
H3:Behavior intention ← Perceived behavior control	0.012	3.802	0.162	***	Yes
H4:Behavior intention ← Knowledge	0.256	3.018	0.152	**	Yes
H4a:Perceived behavior control ← Knowledge	1.487	6.010	0.510	***	Yes
H4b:Attitude ← Knowledge	1.448	2.968	0.109	**	Yes

** *p <0.01,*

*** *p <0.001*.

### Test of Indirect Effect

Each variable was also tested for its direct, indirect, and total effects. As presented in Table 5, the primary care physicians' SN impacted their behavioral intention to provide CCSSs directly and indirectly through attitude and PBC, with a total standardized effect of 0.52. The knowledge level about CCSSs not only affected BI directly but also had a negligible effect on BI indirectly through PBC and attitude (standardized indirect effect = 0.11). Among all variables, SN had the largest effect on behavioral intention to provide CCSSs, with a standardized direct effect of 0.31, followed by attitude, PBC, and related knowledge.

**Table 5:** Results of direct and indirect analysis.

**Path (effects from X to Y)**	**Direct Effect**	**Indirect Effect**	**Total Effect**
Bebavior intention ← Attitude	0.251	0.000	0.251
Behavior intention ← Subjective nrom	0.311	0.208	0.520
Behavior intention ← Perceived behavior	0.162	0.000	0.162
control			
Behavior intention ← Knowledge	0.152	0.110	0.262

## Discussion

This study examined the complicated predictors of primary care physicians' intention of providing CCSSs to rural women. In accordance with the hypotheses based on the TPB and earlier studies, the result of the path analysis test ascertained that primary care physicians' attitude, SN, PBC and knowledge level can all positively affect their intention to perform the CCSSs. This finding indicates that a primary care physician with a favorable attitude, support from significant others, higher perceived power to control the barriers to screening, and sufficient screening knowledge would also have a stronger intent to provide CCSSs to rural women. This finding was consistent with prior TPB-based studies conducted in Saudi Arabia (51), Finland (52), and China (53). This is the first known study to use a theoretical model to assess primary health care physicians' intention to perform CCSSs.

In general, primary care physicians in this study showed a strong intention to provide CCSSs, with an average positive intention rate of 79.2%. The high intention of primary care physicians to provide CCSSs in this study may be due to the new healthcare reform initiated in 2009 by the Chinese government, which aimed at improving the primary care workforce. Research (54) has shown that the primary care system, particularly the maternal and child health system, has since been strengthened. Meanwhile, in 2009, the Chinese government launched the NCCSPRA to provide free CCSSs to eligible rural women. At the same time, some municipal authorities have started to fund and organize local screening projects, and many primary care physicians have since been organized and trained. In addition, most of the participants in this study were women, which is consistent with some previous studies (55, 56) that indicated that CCSSs were still primarily conducted by female physicians. This was partly due to the embarrassment that rural women experienced when facing male physicians during cervical cancer screening.

The attitude toward CCSSs was positively and significantly related to the BI to execute this behavior. A similar finding was revealed by Heena et al. (57) that the health professionals' attitudes toward breast cancer screening can positively influence their decision to adopt this method. Moreover, some researchers (58, 59) have considered AB to be a strong predictor of BI in the TPB model; thus, this may be an effective approach to focus on the benefits of performing CCSSs among primary care physicians. Based on the results of this survey, positive beliefs such as cost savings due to CCSSs, a sense of self-fulfillment and satisfaction were significant motivators for the intention to provide CCSSs. This could be explained by Maslow's hierarchy of needs theory (60), which stipulates that everyone has a desire to be respected. Primary care physicians also need to perceive recognition and a sense of contribution when performing CCSSs. Therefore, strategies should emphasize the positive outcomes of CCSSs work. For example, publicizing successful CCSSs cases and providing financial and material rewards can motivate primary care physicians.

The SN was a fairly good predictor of primary care physicians' intent to provide CCSSs, suggesting that primary care physicians who had a stronger intention to perform screenings work had the support of their colleagues, patients, and leaders. Meanwhile, the SN can not only have a direct effect on the BI to provide CCSSs but can also affect BI through AB and the PBC. This means that support from significant others will ensure that primary care physicians have a more positive attitude toward CCSSs and feel more confident in their CCSSs work. This finding was consistent with some previous studies, research done in Kenya (61) revealed that subjective norms accounted for the greatest variance in primary care physician examination behavior. Galaviz et al. (30) suggested that Mexican physicians' intention to prescribe PA is primarily influenced by their subjective norms of this behavior. This research is somewhat different from a Canadian study (62) that found no significant association between SN and BI in Canadian nurses, perhaps because there is a sociocultural difference between the two countries, and strong social support and less individualism can make Chinese primary care physicians value their family and colleagues' opinions. Among SN, approval from leaders, peers, and patients was a significant determinant of primary care physicians' intentions. Therefore, it is desirable for hospitals to establish an enabling environment in which the implementation of CCSSs is encouraged. Bulletin boards and related cultural products that highlight the advantages of CCSSs can be used to create an ideal atmosphere. It is also important to create a harmonious atmosphere between primary care physicians and rural women. Research (19) has shown that due to poor communication, some rural women in China have negative and distrustful perceptions of primary care physicians and often feel uncomfortable in medical facilities, which may explain the low screening rate among rural Chinese women. Therefore, it is essential that hospitals provide appropriate training to primary care physicians in communicating skills with patients. The government should also take initiatives to improve public awareness of support and participation in CCSSs. In addition, supervisors and senior physicians who accept CCSSs can play an exemplary and prominent role for other primary care physicians.

According to the TPB, PBC was a crucial factor in predicting BI, indicating that lack of time, equipment, and skill training would be a barrier for primary care physicians to perform CCS for rural women. The results of this study concur with several current and past studies (52, 63). Given the disparities in financial and medical resources between urban and rural regions in China, primary care facilities, especially in resource-poor areas, have long been unable to attract and retain experienced, high-quality physicians. Participants in this study scored the lowest in the PBC variable, which revealed that primary care physicians in rural areas still face barriers in their screening work, that can prevent them, as primary care physicians, from performing CCSSs. Research has shown that the majority of rural hospitals do not have sufficient resources and funding to organize CCS for rural women. According to the NCWCH (5), at the county level in China, 41.7% of maternal and children health (MCH) facilities are either in deficit or in a state of a balanced budget, and only 7.2% have equipment for pathological examination. In addition, lack of time, equipment, and skill training has been identified as the main barrier for physicians to provide medical services in Mexico (30), Brazil (64), Canada (65), and Europe (66), posing significant barriers for primary care physicians to provide rural women with qualified CCSSs. Thus, PBC may be a significant predictor of medical services in developed and developing countries. The health authority equipping primary care physicians with the skills and resources on CCSSs would provide a pathway to improve their CCSSs delivery behaviors. Also, an expert panel can be established to assist primary care physicians in resolving CCS problems.

Knowledge of CCSSs was significantly associated with primary care physicians' intentions. It was also a predictor of their attitudes toward PBC. The results of this study indicate that physicians who have more knowledge about CCSSs would have more PBC and a more positive attitude toward CCSSs, as well as greater intent to provide CCSSs to rural women. Overall, primary care physicians in this study demonstrated an adequate level of knowledge about CCSSs. This may be due to the high level of education and lengthy work experience of the participants in this study. Research has revealed that physicians with higher education levels would also have a higher level of knowledge about medical services (39); 64.4% of the primary care physicians in this study had an undergraduate degree or higher. Although the overall level of knowledge in this study was high, there were still some troubling findings: only 47.8% of primary care physicians correctly answered the questions regarding CCS initial screening methods; if primary care physicians lack sufficient knowledge of screening methods, they may give improper advice to women seeking CCSSs. These results demonstrate the importance of improving the knowledge level of primary care physicians, which can be done by providing clinical guidelines regarding CCSSs. Regular lectures and enhanced medical education are also worth pursuing strategies to improve the intentions of primary care physicians to provide CCSSs.

This study had several limitations. First, as this was a cross-sectional study, it was not possible to assess the causal relationships among different factors. More rigorous experiments relating to the intentions of primary care physicians are therefore expected in the future. Second, primary care physicians had a positive attitude toward CCSSs, which may have been caused by social desirability bias. Future research should seek more reliable measures of their attitudes. Third, the study measured the BI of primary care physicians to provide CCSSs rather than actual behavior. While the BI is an important predictor of an individual's behavior, a physician's BI may not necessarily reflect actual CCSSs' behavior. Therefore, primary care physicians' actual behaviors in providing CCSSs should be measured in future studies. The main strength of this study was the large sample size (n = 1,120) and the strong theoretical basis employed. The findings of this study also fill a gap in the literature on the intentions of primary care physicians to provide CCSSs to rural women, which can be used as a reference for future management and intervention.

## Conclusion

This study provided support for the efficacy of TPB and its potential constructs to test predictors of CCS behavior among primary care physicians in rural China. The study concluded that AB, SN, PBC, and knowledge level could be potential determinants in explaining and predicting primary care physicians' intention to provide CCSSs. SN was the strongest predictor of primary care physicians' BI. It can not only affect BI directly but also *via* AB and PBC; thus, it is important that hospitals provide a supportive environment for primary care physicians. Some promising strategies should also be introduced that focus on educating primary care physicians about the value of CCSSs and helping them eliminate barriers to the delivery of CCSSs.

## Data Availability Statement

The raw data supporting the conclusions of this article will be made available by the authors, without undue reservation.

## Ethics Statement

This study's ethical admission was approved by the Ethics Committee of Sir Run Run Hospital, Nanjing Medical University. The grant number is 2019-SR-017. We obtained the oral informed consent from each participant in this survey.

## Author Contributions

YH and ZH: methodology, software, and writing—review and editing. YH, ZH, YS, and YM: writing—original draft preparation. ZH, YS, KC, LL, LW, and YH: investigation and data curation. ZH and YS: visualization. YH: conceptualization, resources, supervision, project administration, and funding acquisition. All authors contributed to the article and approved the submitted version.

## Funding

The study was supported by the National Natural Science Foundation of China (Grant No. 71804074) and the China Medical Board (Grant No: 17-277). The funders had no role in study design, data collection and analysis, decision to publish, or preparation of the manuscript.

## Conflict of Interest

The authors declare that the research was conducted in the absence of any commercial or financial relationships that could be construed as a potential conflict of interest.

## Publisher's Note

All claims expressed in this article are solely those of the authors and do not necessarily represent those of their affiliated organizations, or those of the publisher, the editors and the reviewers. Any product that may be evaluated in this article, or claim that may be made by its manufacturer, is not guaranteed or endorsed by the publisher.
